# Managerial Competencies Engaged in Innovative Actions in Primary Health Care: A Qualitative Study of Brazilian Nurses

**DOI:** 10.1155/2023/8746398

**Published:** 2023-10-13

**Authors:** Iria Barbara de Oliveira, Aida Maris Peres, Rodrigo Almeida Bastos, Mary Casey, Fiona Timmins

**Affiliations:** ^1^Federal University of Paraná, Curitiba, Paraná, Brazil; ^2^University College Dublin, Dublin, Ireland

## Abstract

**Aim:**

To explore the managerial competencies engaged in innovative actions among Brazilian nurses working in primary health care.

**Background:**

The mobilization of managerial competencies favors environments that are in transformation. There is a lack of studies that recognize the managerial skills which influence the implementation of innovative actions in PHC.

**Method:**

A qualitative exploratory descriptive approach was developed in the PHC of a municipality in Brazil. A total of 76 nurses who worked in management and care participated with a semistructured script being applied on innovative actions implemented in the service. The data were processed in IraMuTeQ 0.7 and later analyzed by descending hierarchical classification.

**Results:**

The managerial competencies that influenced the development of innovative actions implemented were as follows: communication and teamwork in planning innovative actions, continuing education for application and implementation, and leadership and people management.

**Conclusions:**

Managerial competencies were used as strategies by nurses to implement innovative actions and contribute to engagement and generating positive results, emphasizing autonomy and the role of nurses as agents of change. *Implications for Nursing Management*. The identified management competencies contribute to directing and sustaining innovative actions, as well as to identifying critical nodes that need support and innovation.

## 1. Background

The triple impact of nursing report proposed by the All-Party Parliamentary Group (APPG) of the United Kingdom in 2016 highlighted the relevant contributions of nursing to improve global health. In addition, the report noted that the role of nursing in health systems allows both the improvement of these systems and the development of nursing skills and knowledge [1, 2]. Thus, based on these reflections, it was possible to suggest that universal health coverage is directly related to the investment in the empowerment of the nursing team. Therefore, the APPG realized that the empowerment of the nursing team makes it possible to contribute to the so-called triple impact: to achieve better health, promote gender equality, and sustain economic growth [3]. In this sense, the different health system contexts can be critical scenarios which enable nurses to fully develop their competencies.

Many nurses often face professional barriers and few opportunities to occupy leadership positions [4]. Among the strategies developed to overcome these barriers were the discussion forums of the “Nursing Now Campaign,” which aimed to improve education and development of the profession, as well as to provide better working conditions, share innovative and successful practices based on scientific evidence, and thus develop competencies [1, 2]. One of the important fields for developing competencies and applying innovations to nursing practice is primary health care. In this health context, nurses develop actions aimed at managing health services, research, and teaching to meet the needs of the health team and the population [5]. These actions are evolved and executed based on various managerial competencies such as communication and planning in the sense of undertaking innovations in the health service and therefore promoting the integral care of the people [6].

Primary health care in Brazil is the gateway to the health system, with nurses acting as health unit managers in this context. Consequently, nurses are responsible for managing material resources and teams, as well as providing comprehensive care to the population [7]. As a result, primary health care in Brazil becomes an important field of observation and development of managerial competencies of nurses as leaders in health care.

On the other hand, evidence within the Brazilian context has shown that nurses face challenges in implementing managerial competencies in their daily work in primary health care [7–9]. These challenges stem from a sense of professional unpreparedness combined with an exhaustive and bureaucratic workload. Thus, in the face of these Brazilian challenges, there is an opportunity to understand managerial competencies in order to promote nurses' engagement.

Thus, managerial competencies are understood as skills exercised by nurses which help in their articulating process in health services [10]. The literature has shown this articulation of services in order to describe the main competencies necessary for good management [11–13]. Therefore, in these studies, it is possible to perceive the focus on managerial elements that can compromise the quality of results or even on specific competencies that enhance results in management in primary care. However, nurses face difficulties in developing these managerial skills, which need to be deepened [11, 13]. The mobilization of internal resources necessary for developing competencies is fundamental for developing innovative strategies during management [14]. Innovative actions in public health, beyond the knowledge about the best skills, result in understanding the difficulties confronted in developing such skills [15]. In this sense, for nursing professionals to reach their full potential in services and the health team, they must develop and improve skills through actions produced in service [3]. Although the nurse has training for managerial practice, it is observed that this role is still in the construction process, which denotes the need for professional qualification and recognition of the difficulties [11].

By recognizing themselves as leaders and managers of health care, nurses will be able to develop innovations in their field of activity based on evidence, the willingness to contribute, and their professional experience [5]. The interface between innovative actions developed by nurses in primary health care (PHC) and their managerial skills will enable developing strategies to support nurses and health care services in the search for organizational results based on innovative actions. The objective of this qualitative study was to explore the managerial competencies engaged in innovative actions among Brazilian nurses working in primary health care.

## 2. Methods

### 2.1. Design

A qualitative exploratory, descriptive approach was used. This approach was chosen to explore the meanings of the managerial experiences of Brazilian nurses in developing innovative activities in PHC. The consolidated criteria for reporting qualitative research (COREQ) tool [16] was used to assess the quality of the research and the trustworthiness of the qualitative approach.

### 2.2. Theoretical Framework

The central results of each cluster were analyzed in light of the theoretical framework of Quinn et al. [17], which highlights the reflection on why different managerial competencies are essential, regardless of the activity or function performed. Therefore, it is relevant that leaders apply various competencies in different activities when thinking about long-term success. Its theory is based on modules which include the following: organizational goals, considering the associated management models; the paradoxes encountered by managers when trying to achieve established goals; and competencies, which the manager can use to transcend or combat these paradoxes. Modules collaborate, create, control, and compete and their managerial competencies can be seen in Table 1.

Quinn et al. [17] observe that different managerial competencies are necessary for effective management. Since some institutions use the model of opposites in their activities, whether innovative or not, meaning that at the same time as they desire adaptable and flexible organizations, managers want them to be stable and controlled. In addition, they want to value and respect employees while also wanting to address the demanding plans and goals. This model is known as the Competing Values Framework [17].

For this study, the focus will only be on managerial competencies. However, when performing actions aimed at collaborating, controlling, competing, and creating, there is a need to integrate several managerial competencies. Thus, this framework has the ability to integrate a diverse set of skills as its central purpose, allowing the manager to act effectively in a constantly changing environment [17]. In addition to acquiring knowledge, the development of competencies requires the behavioral capacity of how to act correctly. Therefore, the manager needs to use and develop them to improve their professional practice because “strategies that are effective in one situation are not necessarily effective in another” ([17], p. 13).

### 2.3. Setting

This study was conducted in a municipality in southern Brazil. The PHC in this municipality has 111 health units characterized as 64 family health strategy units (*Estratégia Saúde da Família*, ESF) and 47 basic units in the traditional model, totalling an estimated population of 550 nurses in primary health care. The traditional basic units present a service model to the people aimed at medical and nursing consultations, with nurses and general practitioners in their team without specific training in family health. The ESF aims to expand the resolution of the services provided, as well as impact the health situation of people and the community, corresponding to a service model offered by a multidisciplinary team of higher and technical levels, usually composed of at least four members: a general practitioner, or specialist in family health, a general nurse or specialist in family health, nursing assistant or technician, and community health workers. Oral health professionals are also added such as dental surgeons, assistants, or technicians in oral health. The reason for selecting this municipality was because it stands out for implementing innovative actions in the Brazilian reality, with these health units being awarded the seal of quality.

### 2.4. Participants and Recruitment

The participants were selected through judgment sampling, which involved the researchers' judgment in choosing the participants who met the appropriate information criteria for the research objectives [18]. Eligible interviewees were nurses with experience in the care or management of health units in the last two years and remained in the position until data collection was completed. Theoretical saturation was employed as a sample closure strategy. Within this approach, the insights provided by new research participants would offer little additional value to the already collected data, thus ceasing to significantly contribute to enhancing the theoretical reflection grounded in the data being gathered [19].

### 2.5. Data Collection

Data collection took place from February to November 2018 through semistructured and audio-recorded interviews. A pilot interview was conducted with a nurse manager and a clinical nurse from a health unit prior to data collection, from which the instrument was improved. The individual interviews were performed by a team of three researchers trained to conduct the interview, two nurses and a fifth-year nursing student. Contact with the participants took place via instant messaging applications and/or e-mail, in which the interviews were previously scheduled. District managers' telephones and electronic addresses were obtained via contact with the institution under study. After contacting the district managers, they indicated the other nurses according to their territories. It should be noted that the instant messaging application is used as a management tool in the institution, and professionals use it as a communication channel with greater efficiency to exchange information.

The researchers introduced themselves and verbally described the research objectives and the data collection process. Data were collected in the work environment of each participant. Each interview lasted between 10 and 40 minutes. The semistructured script prepared for this study provided questions regarding participant characterization, report on an activity considered to be innovative in their work environment, report on the knowledge and information used, how this information was introduced, and the existence of external influence, as well as a description of the adaptation process and the settings. All interviews were transcribed using an audio management application. The transcript quality was verified by the main researcher.

### 2.6. Data Analysis

Data processing took place using a textual analysis software program called IraMuTeQ (*Interface de R pour les Analyses Multidimensionnelles de Textes et de Questionnaires*), version 0.7 alpha 2. It is a free software program developed under the logic of open source, licensed by the GNU GPL (v2), which is anchored in the R software program and in the Python language. Thus, it was possible to organize the data and perform statistical analyses on the combined outcome of the transcribed interviews (textual corpus) and on individual/word tables through the software program by simple and multivariate analyses such as descending hierarchical classification (DHC) [20]. In turn, groups of texts from the combined interviews on the same theme were built and then incorporated into a single text, called the textual corpus. The analyses were carried out through the textual corpus and also through matrices organized in spreadsheets with rows and columns for individuals and words [20].

A DHC presents the relationship between text segment clusters with three lines or more. Furthermore, a set of these text segments divides itself according to their frequency, which enables grouping statistically significant words and qualitative data analysis. Therefore, each corpus presents a text segment (TS) that is considered the central unit of textual analysis illustrating the categories, called clusters in this study. From the corpus, the TS presented in each cluster enables identifying statistically significant words which allow this format of qualitative data analysis ([20], p. 9). First, an exhaustive reading of the TS of each cluster was performed in order to qualitatively understand their meaning. Next, the TSs that best represented each of the clusters were selected to present the results. Accordingly, text segment clusters were obtained in terms of the most frequent words through a dendrogram, which makes it possible to identify the lexical content present in each of the clusters processed by the IraMuTeQ program.

### 2.7. Ethical Considerations

This study was approved by the Research Ethics Committee of the Federal University of Paraná, Brazil, under protocol number 2,157,244.

## 3. Results

The number of participants in this study was 76. All the participants were nurses, 60.5% (*n* = 46) were clinical nurses, and 39.4% (*n* = 30) performed managerial functions at the Municipal Health Department of the municipality under study. It was noted (Table 2) that 40.7% (*n* = 31) were aged between 31 and 40 years, and only 1.3% (*n* = 1) had a doctorate degree, while 53% (*n* = 41) of the nurse managers and 28% (*n* = 21) of clinical nurses had a specialization. Regarding the time in the current position, it was evidenced that the nurse managers who worked from one to five years corresponded to 25% (*n* = 19), and 18% (*n* = 15) had remained in the position from 10 to 15 years. Lastly, time over 15 years of an association with the institution presented 30.2% (*n* = 23) for clinical nurses and 15.7% (*n* = 12) for nurse managers.

**Figure 1:**
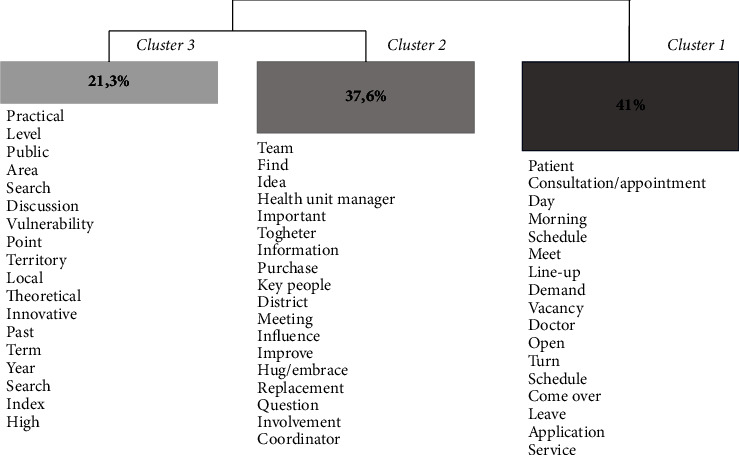
DHC dendrogram with clusters based on the corpus of interviews with clinical nurses and nurse managers.

Data processing from the 76 texts using the IraMuTeQ software program took three minutes and two seconds, resulting in 3,232 TSs with use of 2,595 (80.29%), of which three clusters were generated. Next, DHC was generated through a multivariate statistical analysis of the textual corpus (Figure 1).

All clusters demonstrated the nurses' perception of managerial skills that influenced the development of innovative activities implemented in PHC. It is noteworthy that innovative activities were reported by nurses as not necessarily being created but adapted as innovations according to the reality of the work process. The results pointed to 42 innovative activities.

*Cluster 1*, “communication and teamwork in planning innovative activities,” corresponds to the third column in the dendrogram (Figure 1) and presents text segments which highlight managerial competencies related to improvement of the work process. The planning of innovative activities in the report considered the workflow and the organization form of the teams.

*“[…] the team worked together, […] and she [the local manager] did it now for the whole team, and the whole team commenting on what it was [the innovative activity], how they reached the common goal and we saw their enthusiasm.” (DS 8 Manager).*

From the dendrogram referring to Cluster 1, the planning of activities brought the need to talk about the characteristics of the work process. This demand-based planning consequently enabled nurses to find a new way of demonstrating their role in the health service.

*“Here, in our reality, it is only the nurse who has the application agenda [implanted innovative activity]. Not the nursing technicians, we realize that it is the nurse who provides this service, that the vast majority resolves the issue there.” (HU 12 Manager).*

Thus, it is observed that managerial competencies were fundamental for implementing innovative activities by nurses, both in the scope of management and in care management. It was possible that these skills therefore collaborated for a process of autonomy of the PHC nurse.

*Cluster 2*, “management skills that influenced the role of nurses in innovative activity,” corresponds to the second column in the dendrogram (Figure 1). The text segments show managerial skills: teamwork, communication, continuing education, and leadership. The relevance of the team's knowledge and its involvement in improving the service according to what had been proposed by the implemented innovative activities is noted in the reports.

*“What made it easier, I think, was the involvement and knowledge of the team.” (Clinical 2).*

Difficulties in communication were also evidenced in the text segments of clinical nurses. We observed the difficulty felt in the dialogue and transfer of information from institutional leaders.

*“Currently I see that this dialogue and this exchange have become increasingly difficult, I don't know if it's because of the accumulation. […] so sometimes the way to present this innovation is making some implementation processes difficult.” (Clinical 11).*

Participants reported the challenges of using continuing education as a team engagement tool.

*“It was more knowledge that we got ready; that she [the local manager] brought to us and we began to understand that this process would really make a difference for us.” (DS Manager 4).*

Leadership seemed to emerge as a managerial competence that contributes to the nurse being the main influencer of the team.

*“The first issue is leadership, appropriation of knowledge, belonging. When we say that we want to reach the goal, that we want to serve that community that needs it there.” (DS Manager 2).*

*Cluster 3*, “leadership and people management: fundamentals for applying innovative activities,” corresponds to the first column in the dendrogram (Figure 1) and emphasized leadership and human resource management for managerial skills. The text segment highlighted both competencies as a reference for developing the teamwork process and for the nurse's role as an articulator of this process.

*“The doctor is often not that involved, people who are articulating that go after, inviting, but the nurse is the basis of everything.” (Clinical 26).*

We observed that leadership and people management are directly modulated by the type of population and territory to be worked.

*“They took care of places [referring to the innovative activity: sweeping the territory with ACS and volunteers], for which they collected food to give to people. So, I thought like this: I have this problem, and I don't have human resources, but I know this group that does this work” (HU Manager 5).*

Thus, the innovative activities developed for the vulnerabilities of the assisted people need a strategic vision from the leader to confront the indexes that characterize the population, as well as organizing the teams to carry out the activities. Therefore, local leadership and people management are skills which were perceived as pillars in implementing innovative activities.

*“It was a very interesting and very gratifying moment [implementation of the innovative activity: Saúde Já application], where we had the freedom to manage the patient and to seek worker satisfaction, value the worker, motivate them to recognize their work in the area.” (DS 18 Manager).*

## 4. Discussion

The aim of this qualitative study was to explore the managerial competencies engaged in innovative actions among Brazilian nurses working in primary health care. The findings indicated there was a connection between managerial competencies and the innovative actions developed by nurses in PHC. These innovative actions are evidence of the role of nurses in developing work processes in health services. Thus, valuing nursing actions and creating new strategies for health work improve PHC [14].

As a result, managerial skills, leadership, teamwork, and communication stood out among the three clusters. In this sense, innovative activities, such as those implemented by the participants of this study, go beyond the practices already traditionally performed by nurses. This advance provides the opportunity to reflect and self-assess the work process, as well as to improve their managerial competencies [21]. Therefore, the professional contribution of the nurse as a team leader must generate knowledge, develop skills, and collaborate to implement innovations in the service [22].

It is observed that many performed actions and strategies in PHC are based on the management and care dimensions, whose perspective is to reach beyond the team and service to the community. However, mobilizing some managerial competencies, such as planning, leadership, and people management need to be considered for these to be quality actions [23]. These demands are not always foreseen as job responsibilities, but often arise as demands of the work process. Innovative actions result in transposing an idea from one context to another, with a perspective to improve the work process. Thus, the implementation of innovations is achieved due to the ability of this action to unite people so that together they can execute their best reasoning. In the long term, the organization is expected to become a space where professionals perform innovative activities as a habit, from where all professionals are creators of new ideas and not just the managing leader [17].

Cluster 1 showed innovative activities aimed at improving work processes, such as organizing the service flow and planning and time management being outlined based on communication and teamwork. These are competencies found in both care execution/provision and in the nurse's managerial competencies, further constituting a link between the other managerial competencies [24]. The use of managerial communication competence demonstrated the successful development of innovative activity through dialogue among the team. However, it was up to those involved to use it as a strategy. In practice, communication and teamwork are understood as facilitators of the nurse's leadership process in planning actions [23].

The influence of communication was related to organizing the health service, as it favored consolidating different knowledge held among team members [22, 25], demonstrating that effective communication also requires reflective listening, because when associated, they result in increased interest and trust among team members [17]. As the coordinator of the innovative activity, nurses used communication to negotiate the rational use of various resources with the team to favor the implementation process of the innovative activity. Hence, if the structural dimension of the quality of nurses' work is the basis for executing care actions, communication is observed as the basis for planning structural issues, such as the management of people and material resources.

The institutional barriers of health systems can be better managed in favor of action planning when nurses' communication is previously established and teamwork already has an effective routine. Therefore, the nurse plays the role of the coordinator and planning member of the team's work processes in the context of PHC [26]. In turn, this managerial position contributes to the visibility of their professional role since there is planning in PHC based on the situational diagnosis of this nurse. As a consequence of this visibility, the process of professional autonomy in the services can be built, encouraging the professional to plan, act, and maintain a culture of care quality in PHC as much as possible [5].

In Cluster 2, competence in working in a team was considered particularly challenging, as it means developing a job or activity with motivated professionals focused on the same objective. Therefore, it is not just about having an effective communication process, but constantly dealing with communication difficulties and leadership support. In turn, it is up to the nurse to encourage good teamwork, so that this competence is integrated and continuous as a quality culture in PHC [21], even though communication is considered one of the most challenging skills for new project managers [17]. In this perspective of strengthening the relationship with the team, the nurse develops empowerment in exercising their leadership. There is also improvement in work processes and successful implementation of the innovative activity [24]. As said, the reality of teamwork depends on an effective communication process. When it comes to implementing innovative actions, this communication has constant difficulties. The data of this study converge with what is advocated by the triple impact of nursing when it is observed that management makes it difficult for the nurse leader to share knowledge by following the vertical communication model [4]. In this sense, articulations between professionals can occur informally as long as it is positive, which denotes a good relationship. In addition to involving the team in the activities of PHC services, it is also necessary to integrate the practice of meetings. When communication is safely used, it builds trusting relationships and is considered fundamental for any type of change within the service [17, 27].

In this way, the responsibility of the manager/leader is highlighted in clarifying to each team member their functions given the activities performed. Regardless of their technical or personal skills, which are essential for contributing to the action to be performed, it is relevant that the manager explains what performance is expected from each person on the team to avoid ambiguity and role conflict. Furthermore, the clarity in functions helps the team to understand what to expect from each other. It helps improve communication, which is associated with the collaborate quadrant, that is, attentive listening to members' interests, feedback, and support in conflict resolution [17].

The results demonstrated nurses' difficulties in using the managerial competence in continuing education as a tool for team engagement. As much as everyone was involved, the knowledge about the innovative activity was not built by the team. Despite continuing education being a managerial competence capable of providing services with improved conditions and stimulating the search for knowledge, transforming reality, and qualifying management and care, managerial leaders have the role of guiding and developing the team [17]. This managerial competence still proves to be a difficulty to implement in PHC services [26].

Leadership in Cluster 3 of this study was understood as a pillar for applying innovative activities. Nurses' understanding of the search for knowledge appropriation demonstrates the guarantee of which scientific evidence can effectively support them in their practice [28]. Thus, it is possible to not only ensure leadership and influence in the team but also to optimize the service and care provided, which contribute to improving the results of the implemented actions [10]. Applying innovations requires vision of the processes and critical sense of the results. The nurse's managerial competencies in these dimensions must produce motivation and listen to the team, since the challenge is to be an agent of reality transformation. Nurses have the same perception about how to exercise leadership competence. Thus, the influence exerted on the team contributes to the power figure of the nurse; however, this power occurs due to the relationship of trust that this professional tends to manifest, and not to authoritarianism [10].

Developing and knowing how to share the organisational vision contributes to the organisation's success, as leaders communicate their vision through words and actions. In this way, the team knowing its functions and responsibilities in detail is as essential as the manager's panoramic view [17]. Likewise, institutional leaders converge to the figure of power within the service to nurses, placing them as a protagonist who reflect on their practice and expose their opinion in order to problematize the scenarios discussed in the team. Therefore, the power relationship of healthy leadership is a potential tool to produce innovations fueled by the trust of the PHC team, users, and institutional management [29].

In addition to being considered a managerial competence, leadership is also associated with a cross-cutting theme of the strategic guideline of the region of the Americas. Thus, it also contributes to the objective of strengthening the nursing workforce [30]. In contrast, it is possible to observe some difficulty in developing autonomy in decision-making in certain scenarios, limiting leaders in human resource management [21]. It is therefore important to emphasize the consolidation of nurses' managerial competencies, as they can influence the quality of care for users and their families [15]. Thus, exercising a broad vision of the organization, associated with managerial leadership competence, reflects the concept of visionary leaders. These leaders go beyond managing people and the organization but positively inspire team members, urging them to get involved in the work with extra effort, which results in the consequent likelihood that services will improve [17].

It should be noted that there is still a deficiency in training regarding managerial competencies [31]. However, the link between implementing innovative activities and the managerial competencies developed and used by nurses in PHC demonstrates the commitment by these professionals to the daily improvement of the service provided and the consolidation of PHC attributes.

### 4.1. Limitations

This study is limited due to the analysis of managerial competencies through the report of innovative actions, in which the competencies that were intrinsic in the nurses' speeches were analyzed. Accordingly, it is suggested to search for data beyond innovative actions to expand the analysis of mobilized managerial competencies, as well as in other areas of the health system.

## 5. Conclusion

Essential managerial competencies such as leadership, communication, continuing education, and planning were observed in this study. In turn, these competencies helped in developing innovative activities and consequently collaborated to elevate the professional role of nurses working in PHC, with emphasis on professional autonomy and protagonism.

## 6. Implications for Nursing Management

By reflecting on the managerial competencies developed by nurses in the practice of innovative actions, this study demonstrated the engagement of these professionals in providing an innovative environment in a public sector. Moreover, how much the mobilization of managerial competencies favors management actions was evidenced. In addition to contributing to direct and sustain the proposed innovative actions, they demonstrate that they favor service or care management actions when used as support for practice in identifying critical nodes in the nurse's work process, which need support and innovation. In this perspective, it is reinforced that managerial competencies become facilitating skills to support nurses in directing actions which contribute to their empowerment and to the process of establishing themselves as a recognized and valued profession.

## Figures and Tables

**Table 1:** Managerial competencies according to the model Competing Values Framework—collaborate, create, control, and compete adapted from the study by Quinn et al. [17].

Managerial competencies: collaborate, create, control, and compete	
Modules	Managerial competencies
Module 1: collaborate—create and sustain commitment and cohesion	(i) Understanding yourself and others
	(ii) Communicate honestly and effectively
	(iii) Guide and develop people
	(iv) Manage groups and lead teams
	(v) Manage and encourage constructive conflict

Module 2: control—establish and maintain stability and continuity	(i) Organize information flows
	(ii) Work and manage across roles
	(iii) Plan and coordinate projects
	(iv) Measure and monitor performance and quality
	(v) Encouraging and enabling compliance

Module 3: compete—improve productivity and increase profitability	(i) Develop and communicate a vision
	(ii) Establish goals and objectives
	(iii) Motivate yourself and others
	(iv) Design and organize
	(v) Manage execution and seek results

Module 4: create—promoting change and encouraging adaptability	(i) Using the power and kinetic influence and effectiveness
	(ii) Sponsor and sell new ideas
	(iii) Stimulate and promote innovation
	(iv) Negotiate agreements and commitments
	(v) Implement and sustain change

**Table 2:** Demographic characteristics of clinical nurses and nurse managers.

Demographic characteristics	Clinical nurses	Nurse managers
*Age group*		
21 to 30 years	1	1
31 to 40 years	15	16
41 to 50 years	7	18
51 to 60 years	5	11
61 to 70 years	2	0

*Professional qualification*		
Undergraduate	5	0
Specialist	21	41
Master's	4	4
Doctorate	0	1

*Time in current position*		
0 to 1 year	0	12
1 to 5 years	0	19
5 to 10 years	3	7
10 to 15 years	15	7
15 years or above	12	1

*Time in municipal health department*		
0 to 1 year	0	0
1 to 5 years	0	2
5 to 10 years	12	5
10 to 15 years	11	11
15 years or above	23	12

## Data Availability

The data used to support the findings of this study are available from the corresponding author upon reasonable request.
